# Intravenous iron and SGLT2 inhibitors in iron‐deficient patients with heart failure and reduced ejection fraction

**DOI:** 10.1002/ehf2.14742

**Published:** 2024-03-28

**Authors:** Kieran F. Docherty, John J.V. McMurray, Paul R. Kalra, John G.F. Cleland, Ninian N. Lang, Mark C. Petrie, Michele Robertson, Ian Ford

**Affiliations:** ^1^ BHF Cardiovascular Research Centre School of Cardiovascular and Metabolic Health University of Glasgow Glasgow UK; ^2^ Department of Cardiology Portsmouth Hospitals University NHS Trust Portsmouth UK; ^3^ Faculty of Science and Health University of Portsmouth Portsmouth UK; ^4^ Robertson Centre for Biostatistics University of Glasgow Glasgow UK

**Keywords:** Chronic heart failure, Intravenous iron, Iron deficiency, SGLT2 inhibitor

## Abstract

**Aims:**

To explore the potential interaction between use of SGLT2 inhibitors and the increase in haemoglobin in patients randomized to intravenous iron or the control group in the IRONMAN (Effectiveness of Intravenous Iron Treatment versus Standard Care in Patients with Heart Failure and Iron Deficiency) trial.

**Methods and results:**

This was a post hoc exploratory analysis of the IRONMAN trial which randomized patients with heart failure, a left ventricular ejection fraction (LVEF) ≤ 45% and iron deficiency (transferrin saturation <20% or ferritin <100 μg/L) to open label intravenous ferric derisomaltose or usual care. Of the 1137 randomized patients, 29 (2.6%) were taking an SGLT2 inhibitor at baseline. The mean (SD) change in haemoglobin from baseline at 4 weeks in those taking an SGLT2 inhibitor at baseline was 1.3 (1.2) g/dL in patients randomized to ferric derisomaltose and 0.1 (0.7) g/dL in the usual care group; between‐group difference = 1.0 g/dL (95% CI 0.1, 1.8). The equivalent numbers in the no SGLT2 inhibitor group were 0.6 (0.9) g/dL in those randomized to ferric derisomaltose and 0.1 (0.8) g/dL in the usual care group; between‐group difference = 0.4 g/dL (95% CI 0.3, 1.6); interaction *P* value = 0.10. No patient receiving an SGLT2 inhibitor at baseline developed polycythaemia during follow‐up (defined as haemoglobin >16.5 g/dL [men] or >16 g/dL [women]).

**Conclusions:**

In the IRONMAN trial, there was a trend to a greater increase in haemoglobin with ferric derisomaltose in iron‐deficient patients taking an SGLT2 inhibitor at baseline, as compared with those not taking one.

## Introduction

Iron deficiency is common in patients with heart failure and is associated with anaemia and a poorer prognosis. In patients with heart failure and reduced left ventricular ejection fraction (HFrEF), correction of iron deficiency with intravenous iron preparations improves symptoms, increases exercise capacity and reduces the risk of hospitalization for worsening heart failure.^1^ Accordingly, clinical practice guidelines recommend that patients with HFrEF should have tests for iron deficiency done routinely and receive intravenous iron when indicated.^2^

Recently, there has been increasing interest in the potential interplay between administration of intravenous iron and the use of sodium‐glucose cotransporter 2 (SGLT2) inhibitors. As well as reducing the risk of worsening heart failure and cardiovascular death in patients with heart failure and a broad range of ejection fractions, SGLT2 inhibitors increase haematocrit and haemoglobin, which is thought to be due to either reductions in plasma volume, increased erythropoiesis, or both.^3^, ^4^ Treatment with SGLT2 inhibitors results in changes in biomarkers consistent with improved iron utilization (increased serum transferrin receptor and reduced ferritin, transferrin saturation and hepcidin).^5^ Intravenous iron and SGLT2 inhibitors combined might lead to a rapid increase in haematocrit that could increase the risk of thromboembolic effects.^6^, ^7^ Accordingly, we investigated possible interactions between use of SGLT2 inhibitors and the increase in haemoglobin in patients randomized to intravenous iron or the control group in the IRONMAN (Effectiveness of Intravenous Iron Treatment versus Standard Care in Patients with Heart Failure and Iron Deficiency) trial.

## Aims

To explore the potential interaction between intravenous iron and SGLT2 inhibition, we examined changes in haemoglobin in patients according to baseline use or not of an SGLT2 inhibitor in a post hoc exploratory analysis of the IRONMAN trial.^8^

## Methods

The design, baseline characteristics and main results of the IRONMAN trial have been published.^8^, ^9^ In short, 1137 patients with heart failure, a left ventricular ejection fraction (LVEF) ≤ 45% and iron deficiency (transferrin saturation <20% or ferritin <100 μg/L) were randomized 1:1 to open label intravenous ferric derisomaltose (weight and haemoglobin adjusted dose) or usual care. Use of an SGLT2 inhibitor at baseline was reported by site investigators. Blood samples for haemoglobin levels were collected at baseline, 4 weeks and 4 months. All analyses were performed using SAS version 9.4.

## Results

Of the 1137 randomized patients, 29 (2.6%) were taking an SGLT2 inhibitor at baseline (15/569 [2.6%] in the ferric derisomaltose group and 14/568 [2.5%] in the usual care group). Mean LVEF (31.6% in both groups) and median NT‐proBNP (1470 pg/mL vs. 1717 pg/mL) were similar whether patients were taking an SGLT2 inhibitor or not, whereas a history of diabetes was more common in those taking an SGLT2 inhibitor (79% versus 45%). Mean (SD) haemoglobin at baseline was 11.9 (1.3) g/dL and 12.0 (1.1) g/dL in patients taking or not taking an SGLT2 inhibitor, respectively. Other baseline characteristics according to baseline SGLT2 inhibitor use are summarized in *Table* S1. Among patients randomized to ferric derisomaltose, median baseline dose did not differ according to SGLT2 inhibitor use or not (median 1500 mg in both groups).

**Figure 1:**
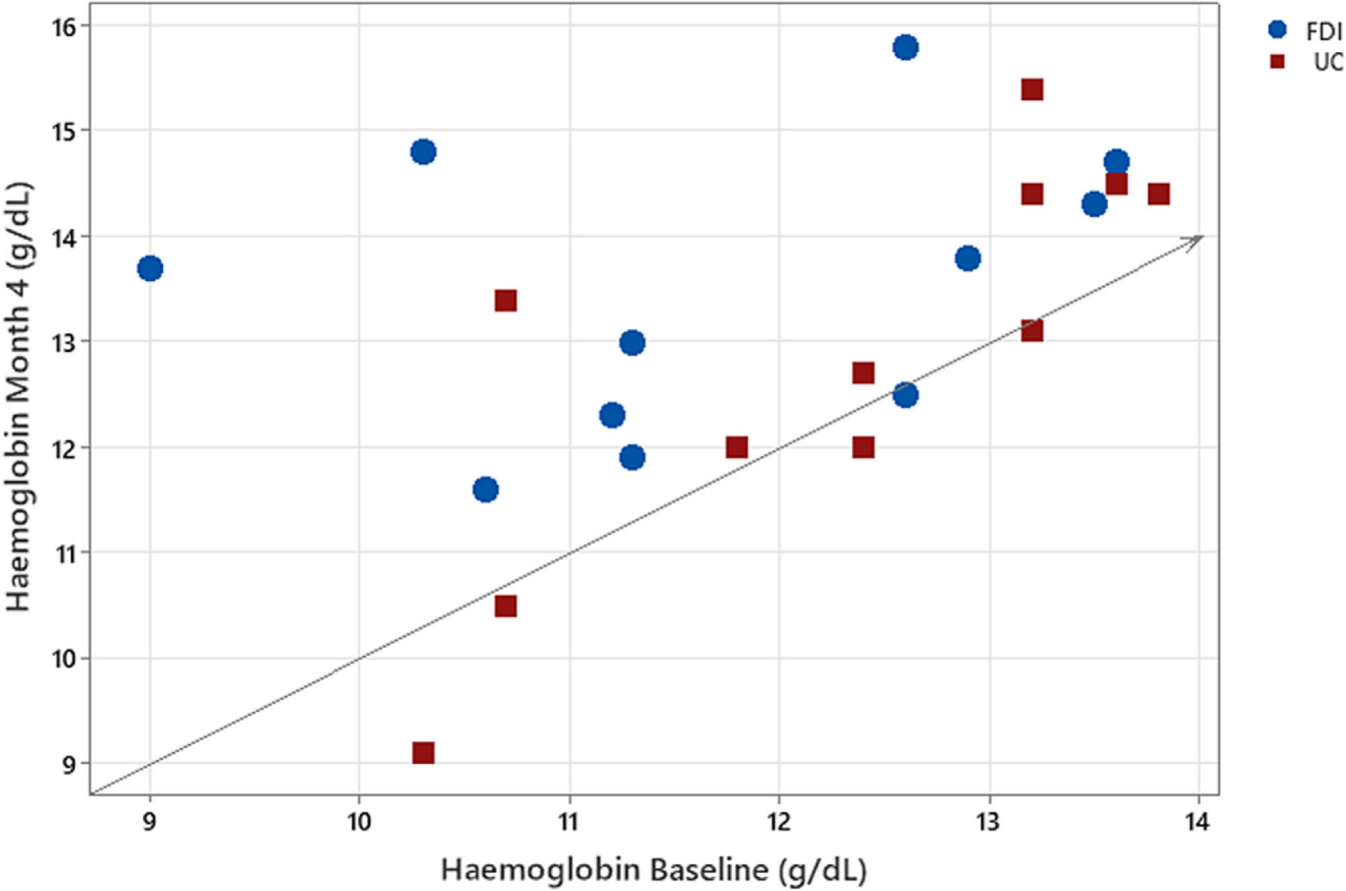
Individual patient changes in haemoglobin at 4 months according to randomized treatment group in patients taking an SGLT2 inhibitor at baseline. FDI, ferric derisomaltose; UC, usual care.

The mean (SD) change in haemoglobin from baseline at 4 weeks in those taking an SGLT2 inhibitor at baseline was 1.3 (1.2) g/dL in patients randomized to ferric derisomaltose and 0.1 (0.7) g/dL in the usual care group; between‐group difference in least square means adjusted for baseline value = 1.0 g/dL (95% CI 0.1, 1.8) (*Table* *1*). The individual patient changes in haemoglobin from baseline at 4 months in those taking an SGLT2 inhibitor at baseline are displayed in *Figure* *1*. The equivalent numbers in the no SGLT2 inhibitor group were 0.6 (0.9) g/dL in those randomized to ferric derisomaltose and 0.1 (0.8) g/dL in the usual care group; between‐group difference = 0.4 g/dL (95% CI 0.3, 1.6); interaction *P* value = 0.10. Similar results were seen at 4 months (interaction *P* value = 0.45) (*Table* *1*). No patient receiving an SGLT2 inhibitor at baseline developed polycythaemia during follow‐up (defined as haemoglobin >16.5 g/dL [men] or >16 g/dL [women]). Among patients not taking an SGLT2 inhibitor at baseline, there was 1 case of polycythaemia among patients randomized to intravenous iron at 4 weeks, and 1 case in each randomized group at 4 months. Among those taking an SGLT2 inhibitor at baseline, two patients randomized to ferric derisomaltose had a fatal or non‐fatal myocardial infarction or stroke, as compared with no events in the usual care group. In patients randomized to ferric derisomaltose, there were no significant differences according to SGLT2 inhibitor use in the change from baseline at 4 weeks or 4 months in ferritin concentrations or transferrin saturation (*Table* *2*).

**Table 1:** Changes in haemoglobin levels according to use of an SGLT2 inhibitor

	SGLT2 inhibitor at baseline			No SGLT2 inhibitor at baseline			
	IV ferric derisomaltose	Usual care	Between‐group difference (95% CI)	IV ferric derisomaltose	Usual care	Between‐group difference (95% CI)	Interaction *P* value
Haemoglobin at baseline (g/dL)	11.7 (1.3) *N* = 15	12.1 (1.3) *N* = 14	‐	12.0 (1.1) *N* = 554	12.0 (1.1) *N* = 554	‐	‐
Change in haemoglobin at 4 weeks (g/dL)	1.3 (1.2) *N* = 13	0.1 (0.7) *N* = 11	1.0 (0.1, 1.8)	0.6 (0.9) *N* = 514	0.1 (0.8) *N* = 489	0.4 (0.3, 0.6)	0.10
Change in haemoglobin at 4 months (g/dL)	1.8 (1.6) *N* = 11	0.6 (1.1) *N* = 11	1.0 (−0.2, 2.3)	0.8 (1.3) *N* = 439	0.2 (1.2) *N* = 418	0.6 (0.4, 0.8)	0.45

All data presented as mean (standard deviation) unless indicated otherwise. Between‐group difference represents a difference in least square means adjusted for baseline values.

CI, confidence interval; IV, intravenous; SGLT2, sodium‐glucose cotransporter 2.

**Table 2:** Change in iron biomarkers according to SGLT2 inhibitor use in the intravenous ferric derisomaltose group

	SGLT2 inhibitor	No SGLT2 inhibitor	Between‐group difference (95% CI)
Change from baseline			
Ferritin (Week 4), μg/L	338 (315) *n* = 13	444 (239) *n* = 512	−106 (−239, 27)
Ferritin (Month 4), μg/L	248 (216) *n* = 11	276 (173)	−28 (−132, 77)
TSAT (Week 4), %	15.9 (10.3) *n* = 13	13.2 (9.6) *n* = 491	2.7 (−2.6, 8.1)
TSAT (Month 4), %	16.5 (10.1) *n* = 10	11.6 (10.7) *n* = 422	4.9 (−1.8, 11.6)

All data presented as mean (standard deviation) unless indicated otherwise.

CI, confidence interval; SGLT2, sodium‐glucose cotransporter 2; TSAT, transferrin saturation.

## Discussion

To our knowledge, these are the first randomized trial data providing insights into the treatment of iron deficiency with intravenous iron in patients with HFrEF taking an SGLT2 inhibitor, although any conclusions are limited by the small number of patients taking an SGLT2 inhibitor at baseline.

In the DAPA‐HF (Dapagliflozin and Prevention of Adverse‐Outcomes in Heart Failure) trial, the presence of iron deficiency at baseline did not attenuate the benefits of dapagliflozin on clinical outcomes.^5^ A biochemical profile consistent with iron deficiency (low ferritin and transferrin saturation) occurred more frequently during follow‐up in patients randomized to dapagliflozin, consistent with an increased iron demand due to stimulation of erythropoiesis by SGLT2 inhibitors.^5^ Furthermore, the reduction in plasma hepcidin with SGLT2 inhibition is consistent with an improved capacity for iron absorption and increased mobilization of iron from sequestered stores. Giving intravenous iron to patients who have or develop iron deficiency on an SGLT2 inhibitor might augment their haematological effects. This could lead to greater benefits on symptoms, exercise capacity and outcomes. On the other hand, increases in haematocrit induced by erythropoiesis stimulating agents have increased the risk of thrombo‐embolic events.^10^

Previous, non‐randomized reports have suggested a greater haematological response to intravenous iron in patients taking an SGLT2 inhibitor as compared with those not.^11^ Concerns have been raised that administration of intravenous iron in patients taking an SGLT2 inhibitor may cause myocardial iron overload and increase the risk of polycythaemia.^6^, ^7^ However, in DAPA‐HF, the increase in haemoglobin with dapagliflozin tended to be smaller in patients who were iron deficient at baseline, raising the question of whether intravenous iron administration may help increase the benefits of SGLT2 inhibitors in iron deplete patients.^5^ In the IRONMAN trial, patients receiving an SGLT2 inhibitor at baseline would likely have been taking this medicine for several months before randomization but, nonetheless, most remained anaemic; whilst intravenous iron increased haemoglobin it generally did not normalize it. Although the increase in haemoglobin tended to be greater among patients taking SGLT2 inhibitors at baseline, no case of polycythaemia was observed in the first 4 months. Further evidence regarding any interaction between SGLT2 inhibitors and intravenous iron should soon be provided by the HEART‐FID (Ferric Carboxymaltose in Heart Failure With Iron Deficiency) trial (ClinicalTrials.gov identifier NCT03037931).^12^

## Conclusions

In conclusion, in this post hoc exploratory analysis of the IRONMAN trial, there was a trend to a greater increase in haemoglobin with intravenous ferric derisomaltose in iron‐deficient patients with heart failure taking an SGLT2 inhibitor. Any conclusions are limited by the small number of patients taking SGLT2 inhibitors.

## Funding

IRONMAN was an investigator‐initiated trial, designed by members of the TSC (ClinicalTrials.gov identifier: NCT02642562) and funded by the British Heart Foundation (grant award CS/15/1/31175). Pharmacosmos A/S provided and distributed ferric derisomaltose and made an additional contribution to research costs. Drs McMurray, Petrie and Cleland are supported by the British Heart Foundation Centre of Research Excellence Grant RE/18/6/34217.

## Conflict of Interest

KFD reports that his employer, the University of Glasgow, has been remunerated by AstraZeneca for work relating to clinical trials. He has received speaker's honoraria from AstraZeneca, Pharmacosmos and Radcliffe Cardiology, has served on an advisory board for Us2.ai and Bayer AG, served on a clinical endpoint committee for Bayer AG, and has received grant support from Boehringer Ingelheim, Roche Diagnostics and AstraZeneca (paid to his institution). JJVM has received support from a British Heart Foundation Centre of Research Excellence Grant RE/18/6/34217 and the Vera Melrose Heart Failure Research Fund; has received payments through Glasgow University from work on clinical trials, consulting, and other activities from Amgen, AstraZeneca, Bayer, Cardurion, Cytokinetics, GlaxoSmithKline, KBP Biosciences, and Novartis; has received personal consulting fees from Alnylam Pharma, Bayer, Bristol Myers Squibb, George Clinical PTY Ltd, Ionis Pharma, Novartis, Regeneron Pharma, and River 2 Renal Corporation; has received personal lecture fees from Abbott, Alkem Metabolics, AstraZeneca, Blue Ocean Scientific Solutions, Ltd, Boehringer Ingelheim, Canadian Medical and Surgical Knowledge, Emcure Pharma Ltd, Eris Lifesciences, European Academy of CME, Hikma Pharmaceuticals, Imagica Health, Intas Pharma, J.B. Chemicals and Pharma Ltd, Lupin Pharma, Medscape/Heart.Org,

ProAdWise Communications, Radcliffe Cardiology, Sun Pharma, The Corpus, Translation Research Group, and Translational Medicine Academy; and is a director of Global Clinical Trial Partners Ltd. PRK reports research grants from British Heart Foundation and Pharmacosmos; consulting fees from Ackea, Amgen, Boehringer Ingelheim, Pharmacosmos, Servier, and Vifor Pharma; payment for lectures from AstraZeneca, Bayer, Novartis, Pfizer, Pharmacosmos, and Vifor Pharma; support for attending meetings from Pharmacosmos; is a data safety monitoring board member for the STOP‐ACE trial; and has served as Chair of the British Society for Heart Failure. JGFC reports research grants from Amgen, Bayer, Bristol Myers Squibb, British Heart Foundation, Johnson & Johnson, Medtronic, Myokardia, Pharmacosmos, Pharma Nord, and Vifor Pharma; payment for lectures from Abbott, Amgen, AstraZeneca, Boehringer Ingelheim, Innolife, NI Medical, Novartis, Servier, and Torrent; support for attending meetings from Boehringer Ingelheim and Pharmacosmos; is a data safety monitoring board member for Idorsia and Medtronic; has stock with Heartfelt Limited; and has been provided with equipment by Heartfelt Limited and NI Medical. NNL reports research grants from AstraZeneca, Boehringer Ingelheim, British Heart Foundation, and Roche Diagnostics; consulting fees from AstraZeneca; payment for lectures from Novartis and Roche Pharma; and is on a data safety monitoring board or advisory board for Pharmacosmos. MCP reports research grants from AstraZeneca, Boehringer Ingelheim, Boston Scientific, Medtronic, Novartis, Novo Nordisk, Roche, and SQ Innovations; consulting fees from AbbVie, AstraZeneca, Bayer, Boehringer Ingelheim, Cardiorentis, Novartis, Novo Nordisk, Pharmacosmos, Siemens, and Takeda; payment for lectures from AstraZeneca, Boehringer Ingelheim, Novartis, Novo Nordisk, Pharmacosmos, Siemens, and Vifor Pharma; support for attending meetings from AstraZeneca; is a data safety monitoring board or advisory board member for AstraZeneca, Boehringer Ingelheim, Novo Nordisk, Pharmacosmos, Teikoku, and Vifor Pharma; and is a director of Global Clinical Trials Partners. MR, and IF report research grants from British Heart Foundation and Pharmacosmos.

## Supporting information


**Table S1:** Baseline characteristics according to SGLT2 inhibitor use.
