# Supplementary material for: Intravenous iron and SGLT2 inhibitors in iron‐deficient patients with heart failure and reduced ejection fraction
Source: ESC Heart Fail. 2024 Mar 28;11(4):1875–9. doi: 10.1002/ehf2.14742 (PMC11287326; doi:10.1002/ehf2.14742)
Supplement: Supplementary file 1 — Table S1: Baseline characteristics according to SGLT2 inhibitor use. [file EHF2-11-1875-s001.docx]

**Supplementary Table 1: Baseline characteristics according to SGLT2 inhibitor use**

|  | **SGLT2 inhibitor (n=29)** | **No SGLT2 inhibitor (n=1108)** |
| --- | --- | --- |
| Age, years | 70.8 (67.3, 74.4) | 73.5 (66.9, 79.5) |
| Male | 20 (69) | 817 (74) |
| Female | 9 (31) | 291 (26) |
| BMI, kg/m^2^ | 29.0 (24.9-34.2) | 28.4 (24.7-32.5) |
| Recruitment context |  |  |
| Admitted to hospital for heart failure and expected to survive to discharge | 6 (21) | 158 (14.3) |
| Admitted to hospital for heart failure within past 6 months | 6 (21) | 202 (18) |
| Outpatient with raised natriuretic peptide concentration | 17 (59) | 748 (68) |
| New York Heart Association functional classification |  |  |
| II | 18 (62) | 630 (57) |
| III | 11 (38) | 457 (41) |
| IV |  | 21 (2) |
| Heart rate, beats per min | 76 (65, 87) | 69 (60, 79) |
| Systolic blood pressure, mm Hg | 121 (112, 133) | 119 (106, 132) |
| Left ventricular ejection fraction | 33 (25, 40) | 34 (25, 37) |
| Principal cause of heart failure |  |  |
| Ischaemic | 17 (59) | 630 (57) |
| Non-ischaemic | 9 (31) | 364 (33) |
| Unknown | 3 (10) | 114 (10) |
| Medical history |  |  |
| Hospital admission for heart failure | 17 (59) | 644 (58) |
| Atrial fibrillation | 14 (48) | 520 (47) |
| Acute coronary syndrome | 14 (48) | 563 (51) |
| Hypertension | 19 (66) | 593 (54) |
| Diabetes | 23 (79) | 498 (45) |
| Device therapy |  |  |
| Implantable cardioverter- defibrillator | 4 (14) | 324 (29) |
| Cardiac resynchronization therapy | 3 (10) | 240 (22) |
| Haemoglobin, g/dL |  |  |
| Mean | 11.9 (1.3) | 12.0 (1.1) |
| Median | 11.8 (11.1, 12.9) | 12.1 (11.2, 12.8) |
| Estimated glomerular filtration rate calculated by Chronic Kidney Disease Epidemiology Collaboration, mL/min per 1·73 m² | 53.6 (40.9, 61.0) | 51.3 (38.0, 68.4) |
| Heart failure medication |  |  |
| Loop diuretic | 18 (62) | 908 (82) |
| Angiotensin-converting enzyme inhibitor | 6 (21) | 546 (49) |
| Angiotensin receptor blocker | 6 (21) | 546 (49) |
| Sacubitril-valsartan | 12 (41) | 228 (21) |
| Beta-blocker | 28 (97) | 981 (89) |
| Mineralocorticoid receptor antagonist | 15 (52) | 617 (56) |
| Digoxin | 8 (28) | 127 (11) |

Data provided as median (interquartile range) and n (%) unless specified otherwise.
